# Estimating the Instantaneous Screw Axis and the Screw Axis Invariant Descriptor of Motion by Means of Inertial Sensors: An Experimental Study with a Mechanical Hinge Joint and Comparison to the Optoelectronic System

**DOI:** 10.3390/s20010049

**Published:** 2019-12-20

**Authors:** Andrea Ancillao, Maxim Vochten, Erwin Aertbeliën, Wilm Decré, Joris De Schutter

**Affiliations:** 1 Robotics Research Group, Dept. of Mechanical Engineering, KU Leuven, 3001 Leuven, Belgium; maxim.vochten@kuleuven.be (M.V.); erwin.aertbelien@kuleuven.be (E.A.); wilm.decre@kuleuven.be (W.D.); joris.deschutter@kuleuven.be (J.D.S.); 2 Flanders Make, Core Lab ROB, KU Leuven, 3001 Leuven, Belgium

**Keywords:** helical axis, inertial sensors, instantaneous screw axis, invariant descriptor, MIMU, rotation axis

## Abstract

The motion of a rigid body can be represented by the instantaneous screw axis (ISA, also known as the helical axis). Recently, an invariant representation of motion based on the ISA, namely, the screw axis invariant descriptor (SAID), was proposed in the literature. The SAID consists of six scalar features that are independent from the coordinate system chosen to represent the motion. This method proved its usefulness in robotics; however, a high sensitivity to noise was observed. This paper aims to explore the performance of inertial sensors for the estimation of the ISA and the SAID for a simple experimental setup based on a hinge joint. The free swing motion of the mechanical hinge was concurrently recorded by a marker-based optoelectronic system (OS) and two magnetic inertial measurement units (MIMUs). The ISA estimated by the MIMU was more precise, while the OS was more accurate. The mean angular error was ≈2.2° for the OS and was ≈4.4° for the MIMU, while the mean standard deviation was ≈2.3° for the OS and was ≈0.2° for the MIMU. The SAID features based on angular velocity were better estimated by the MIMU, while the features based on translational velocity were better estimated by the OS. Therefore, a combination of both measurements systems is recommended to accurately estimate the complete SAID.

## 1. Introduction

The estimation of the instantaneous screw axis (ISA), also known as twist axis, helical axis or axis of rotation [1], plays a notable role in the biomechanical analysis of human joints. The ISA and its motion is strongly related to the functionality of the joint, the healthiness of the ligaments, and therefore, to the overall performance of the motor act [2].

The ISA is based on the Mozzi–Chasles’ theorem that defines a general rigid-body displacement as a translation along an axis plus a rotation about the same axis [3,4]. The general screw displacement for a rigid body can be formulated in terms of points and lines representing some finite displacements by means of screw displacement pairs [5]. Such a representation for motion was widely adopted in the fields of robotics and motion analysis as it represents a convenient way to describe and manipulate trajectories and the relative motion between rigid bodies, such as the consecutive segments of a robot manipulator [6,7,8]. Another example is a recent study where the ISA representation was exploited to determine the twist and the wrench acting on a vehicle suspension system [9].

The motion of a rigid body can be recorded by means of markers attached to the rigid body itself [10], and in general, three non-collinear markers are enough to reconstruct the rotation axis of a rigid-body [11]. However, it was proven that redundant marker trajectories may help to reduce the influence of noise and artefacts so that the localization procedure is improved [10,12]. In typical in-vivo motion capture, the measurements are affected by sensor noise and soft tissue artefacts; thus, the determination of the ISA becomes less accurate [13,14]. The ISA is generally uniquely defined, except when there is no motion or when the motion consists of a pure translation [6].

Some human joints, such as the knee and the elbow, can be modelled as a hinge as a first approximation [11]. Knowing the direction and location of the ISA, which represents the relative motion between two body segments, may help in such cases to better model the joint, the analysis of pathological patterns and the design and placement of prostheses [15,16].

A method for the in-vivo measurement of the position and orientation of the ISA for the elbow was proposed by Stokdijk et al. [15]. It was based on the measurement of the linear and angular velocity of the two body segments, and it was proven to be reasonably accurate for the functional estimation of the elbow axis, especially for the design and testing of endoprostheses [15].

A more recent work exploited the ISA to develop a model of the human knee joint named “force closure mechanism” [17]. This method expresses the interaction dynamics between two adjacent rigid bodies with respect to the ISA which is obtained from the velocity state. Such a representation can work as an inverse dynamic model for the in-vivo estimation of internal contact forces. The strength of this method lies in the functional estimation of the knee axis instead of a geometric one; thus, the inverse dynamics can be evaluated more accurately with respect to the dominant degree of freedom and independently from the chosen coordinate systems [17]. In order to be applied reliably, this method requires a solid and robust computation of the ISA.

To improve the computation of the ISA, an in-vivo study involving markers fixed directly to the bone trough intracortical pins was proposed in [18]. This approach allowed the accurate study of the knee ISA during a running task with respect to the tibial frame and demonstrated that the human knee does not behave like a perfect hinge. The variation observed in the ISA direction and origin were due to the anatomy of the knee and not due to measurement noise or soft tissue artefact [18]. However, such a method, being highly invasive, cannot be adopted in the clinical practice. For this reason, research studies are required to: (i) reduce inaccuracies in marker data in a non-invasive way and (ii) increase accuracy in the computation of the knee ISA.

In addition to the ISA itself, an invariant representation of the relative rigid-body motion can be defined based on a set of ISA parameters. Such an invariant description of motion, namely, the screw axis invariant descriptor (SAID), is a method that was recently introduced in the literature [19,20] with the aim to provide a coordinate-free representation of the motion to be analysed. E.g., in robotics, such a representation allowed the generalization and generation of motor trajectories based on demonstrated motion [21]. The SAID consists of six scalar functions of time that are independent of the coordinate system, CS, chosen to represent the motion, and it is commonly presented as a set of six curves [19,21]. More in detail, the invariant representation is independent from: (i) the body-attached CS representing the position and orientation and (ii) the absolute CS chosen for recording the motion. The first three scalar features represent the angular velocity-based invariants (*ω_1_, ω_2_, ω_3_*), while the last three features represent the translational velocity-based invariants (*v_1_, v_2_, v_3_*) [19]. The invariants *ω_1_* and *v_1_* represent, respectively, the angular velocity of rotation around the ISA and the linear translation velocity along the ISA. The other invariants *ω_2_*, *ω_3_, v_2_* and *v_3_* describe the rotational and translational motion of the ISA itself. The SAID is analytically defined by higher-order trajectory derivatives, and therefore, it is very sensitive to noise, especially when there is a pure translation or a fixed rotation. The most affected invariants are *ω_2_, ω_3_, v_2_* and *v_3_*, which become undefined when the previous invariants are close to zero [19], and, in general, it is well known that differential kinematics are strongly affected by noise and other disturbances [10,13,19]. These effects are more noticeable when using optoelectronic systems, where angular and linear velocities need to be indirectly estimated from position trajectories. This problem may be overcome by using magnetic inertial measurement units (MIMUs), that are becoming popular in motion analysis for their portability, relatively low cost and increasing accuracy of measurement [22,23,24]. Furthermore, the MIMUs allow the direct measurement of the angular velocity, offering the opportunity to increase the accuracy in the estimation of the ISA axis.

Before studying the ISAs and the SAIDs of anatomical joints, the performance of the sensors on in-vitro controlled experiments has to be examined. Thus the aims of this work were: (i) to implement a setup for estimating the ISA of an artificial hinge by means of inertial sensors; (ii) to compute the screw axis invariant descriptor for the hinge motion; (iii) to comparatively examine the outputs and performance of optoelectronic system (OS) and MIMU systems.

This work is meant as a preliminary study on the ISA axis computation and its invariant parameters, aimed to lay the foundation for future works on human subjects and in-vivo measurement of the knee ISA.

## 2. Materials and Methods

### 2.1. Equipment

An inertial motion capture system and an optoelectronic system (OS) were used concurrently in this study. 

The OS was a Vicon MX^®^ (Vicon Motion Systems, Oxford, UK) equipped with 10 cameras. The sampling frequency was 100 Hz. The system was calibrated prior to every session according to the manufacturer’s instructions. The RMS error in marker reconstruction was ≈0.5 mm for a calibrated volume of about 4 m^3^. Reconstruction of marker trajectories, labelling and general preprocessing was obtained by the software Vicon Nexus 2.8 (Vicon Motion Systems, Oxford, UK).

The inertial motion capture system was an MTw Awinda^®^ (Xsens Technologies B.V., Enschede, the Netherlands) composed of a wireless receiver and two MIMU units wirelessly connected and synchronized to the receiver. The sampling frequency was 100 Hz. Data was recorded by means of the MT-Manager software (Xsens Technologies B.V., Enschede, the Netherlands). Before the experiments, the sensors were warmed up for ≈15 min and then their heading output was reset while keeping them distant from metallic objects. 

As the two systems used the same sampling frequency, no resampling was needed. The data from the two systems were independently recorded and the datasets were synchronized during the post-processing by means of the cross-correlation function applied between the first component of the angular velocities. All the data processing was implemented in MATLAB (The MathWorks, Natick, MA, USA). The data were recorded in the Motion Analysis Laboratory of the Department of Movement Sciences, KU Leuven University, Leuven, Belgium.

### 2.2. Experimental Setup

**Figure 1:**
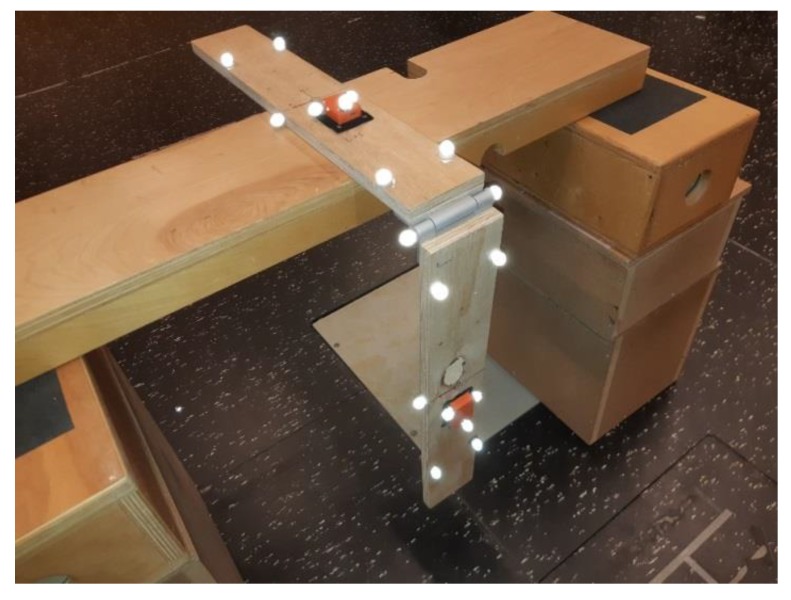
Experimental setup of the artificial hinge equipped with optoelectronic system (OS) reflective markers and magnetic inertial measurement unit (MIMU) sensors (orange).

A mechanical hinge joint was designed as shown in Figure 1. The system was composed of two plywood bars connected by a commercial aluminium hinge. The motion of the hinge was assumed to be a one degree of freedom rotation along the geometrical axis. Each bar was instrumented with a MIMU sensor rigidly attached to the plywood by means of Velcro straps. This ensured no relative motion between the sensor and the rigid body. The overall setup was designed in order to mimic the inertial recording of human motion; e.g., for the knee flexion, two sensors were rigidly fixed to the hip and to the shank [25].

A marker protocol was designed ad hoc for tracking the kinematics of the system. Each bar was equipped with five reflective markers (10 mm diameter) placed at the corners of the bar and one on the side, as shown in Figure 1. Two markers were placed at the extremities of the hinge, centred on the rotation axis. Three additional markers were rigidly attached on each MIMU sensor.

The designed system was placed in the centre of the calibrated volume of the OS, with the upper bar firmly fixed and the lower bar left free to swing. The resting position was with the hinge joint at ≈90° (Figure 1).

In the dynamic trials, the lower bar was raised and immediately released, in order to obtain natural and unconstrained oscillations around the joint axis. The trial was repeated five times. The motion was concurrently recorded by means of the two systems.

### 2.3. Data Processing

The marker trajectories were reconstructed as *x*, *y*, *z* Cartesian coordinates with respect to the global coordinate system CS {0} by means of the Vicon Nexus™ software (Vicon Motion Systems, Oxford, UK). Pre-processing included a moving average smoothing filter, with a window of 10 samples, to reduce sensor noise [26] and spline interpolation to fill possible gaps in the trajectories [27].

**Figure 2:**
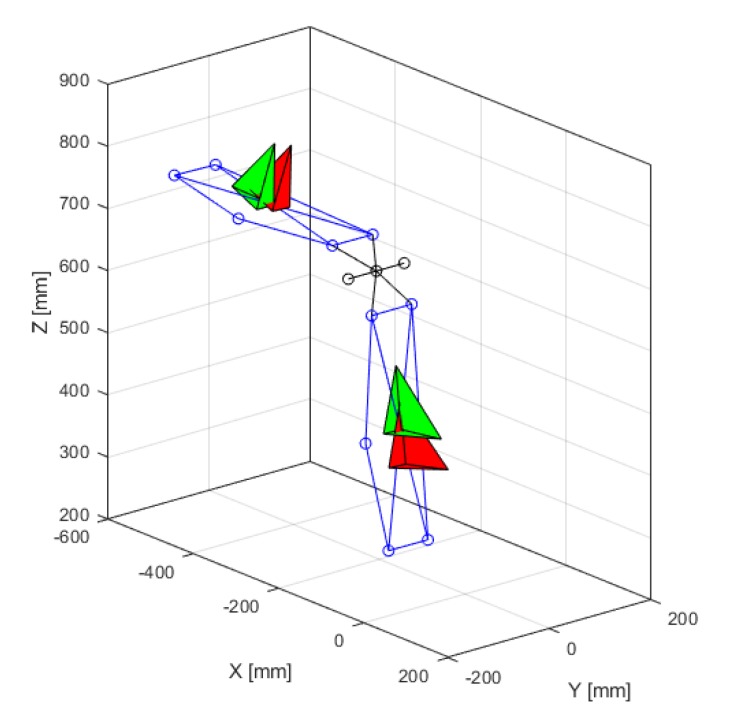
Reconstruction of the hinge bars and their coordinate systems (CS). Green: CS built based on OS markers. Red: CS built based on inertial data. The short line on the tetrahedrons represents the z-axis. The x-axis points out of the surface; the y-axis points upwards.

A local CS was defined for each bar as follows (Figure 2):

- Origin: midpoint of the four markers at the corners of the bar.
- *x*-axis: unit vector normal to the plane defined by the markers at the corners of the bar.
- *z*-axis: unit vector in the direction of the short edge of the bar, pointing to the right, normal to the *x*-axis.
- *y*-axis: cross product between *z*-axis and *x*-axis.

The CSs were defined with respect to the world frame CS {0} and assembled using an optimal localization procedure, that took advantage of the redundant marker information to reduce the errors [12]. The orientations of the two bars were mathematically represented by their rotation matrices: R01OS, i.e., the orientation of bar 1 with respect to 0 measured by means of the OS; and R02OS, i.e., the orientation of bar 2 with respect to 0 measured by means of the OS.

(1) R01OS=(i01xj01xk01xi01yj01yk01yi01zj01zk01z) 

(2) R02OS=(i02xj02xk02xi02yj02yk02yi02zj02zk02z)

The matrices have as columns the ***i*, *j*** and ***k*** unit vectors of the respective CSs (Equations (1) and (2)). The defined reference systems for the two bars are depicted in Figure 2.

In order to allow the comparison of the ISA axes computed by the two systems, it was necessary to align the world CS of the MIMU to the world reference frame of the OS. For this purpose, the markers applied on each MIMU were exploited to define a local CS for each sensor. This CS were defined coherently with the built-in CS of the MIMUs. By means of this procedure, the orientation measured by the MIMU could be referenced to the ground CS of the OS. As a further step, the CS of each MIMU sensor was rotated to match the orientation of the reference system of the bars; i.e., with the *z*-axis parallel to the geometrical rotation axis of the hinge. The rotation matrices representing the orientation of the MIMUs were reconstructed from inertial data by means of the proprietary data fusion algorithm implemented in the MIMU MT-Manager software. It is important to remark that the rotational parts of the CS of the sensors were based on the inertial data only, and, although their orientations were subsequently referenced to the ground CS of the OS, their definition was kept independent from the CS of the bars defined by the OS. The origin of the inertial CS was defined based on marker data, as the midpoint of the three markers placed on the sensor.

The reference systems of the MIMUs, expressed in CS {0}, are depicted in Figure 2 and were mathematically represented using rotation matrices: R01IMU and R02IMU, which were analogous to the matrices represented in Equations (1) and (2).

By taking advantage of the CSs of the bars, CS {1} and CS {2}, the *z*-*y-x* Euler angles of the joint were computed for the relative motion of CS {2} with respect to CS {1}. The relative ISA was computed based on the method proposed by [15] in terms of the orientation of the ISA axis and its origin point; i.e., the centre of rotation. Given the angular velocity **ω** of CS {2} with respect to CS {1} and given p, the position vector (origin) of CS {2} with respect to CS {1}, it is defined as follows:

(3) $$\omega =\;\sqrt{\omega^{T}\omega }$$

(4) $$n=\frac{\omega }{∥\omega ∥}\;$$

(5) $$s=p+\frac{\omega \times \dot{p}}{{∥\omega ∥}^{2}},$$

where ***n*** is the unit vector representing the direction of the ISA axis, and ***s*** is the position vector for the ISA with respect to CS {1}. Both quantities were transformed to CS {0} for graphical visualization and further processing.

In addition to the ISA, a complete set of invariant motion features, based on the ISA, was computed using the closed-form equations (Equations (6)−(11)). The sign of the features depends on the orientation of the ISA [19].

(6) $$\omega_{1}=\pm ∥\omega ∥$$

(7) $$v_{1}=\pm \frac{v\cdot \omega }{∥\omega ∥}$$

(8) $$\omega_{2}=\pm \frac{\omega \times \dot{\omega }}{∥\omega \times \dot{\omega }∥}\cdot \frac{\omega \times \dot{\omega }}{{∥\omega ∥}^{2}}$$

(9) $$v_{2}=\pm \frac{\omega \times \dot{\omega }}{∥\omega \times \dot{\omega }∥}\cdot \frac{\left(\dot{\omega }\times v+\omega \times \dot{v}\right)\cdot {∥\omega ∥}^{2}-2\left(\omega \cdot \dot{\omega }\right)\cdot \left(\omega \times v\right)}{{∥\omega ∥}^{4}}$$

(10) $$\omega_{3}=\pm \frac{∥\left(\omega \times \dot{\omega }\right)\times \left(\omega \times \ddot{\omega }\right)∥}{{∥\omega \times \dot{\omega }∥}^{2}}$$

(11) $$v_{3}=\mp \frac{\left[\dot{\omega }\times \left(\omega \times \dot{\omega }\right)+\omega \times \left(\omega \times \ddot{\omega }\right)\right]\cdot \left[{∥\omega ∥}^{2}\cdot \left(\dot{\omega }\times v+\omega \times \dot{v}\right)-2\left(\omega \cdot \dot{\omega }\right)\cdot \left(\omega \times v\right)\right]}{{∥\omega ∥}^{3}\cdot {∥\omega \times \dot{\omega }∥}^{2}}\;\mp \frac{\left[\omega \times \left(\omega \times \dot{\omega }\right)\right]\cdot \left[{∥\omega ∥}^{2}\cdot \left(\ddot{\omega }\times v+2\dot{\omega }\times \dot{v}+\omega \times \ddot{v}\right)-2\left({∥\dot{\omega }∥}^{2}+\omega \cdot \ddot{\omega }\right)\cdot \left(\omega \times v\right)\right]}{{∥\omega ∥}^{3}\cdot {∥\omega \times \dot{\omega }∥}^{2}}\;\;\pm \left[\frac{3}{2}\cdot \frac{\omega \cdot \dot{\omega }}{{∥\omega ∥}^{2}}+\frac{\left(\omega \times \dot{\omega }\right)\cdot \left(\omega \times \ddot{\omega }\right)}{{∥\omega \times \dot{\omega }∥}^{2}}\right]\cdot \frac{\left[\omega \times \left(\omega \times \dot{\omega }\right)\right]\cdot \left[{∥\omega ∥}^{2}\cdot \left(\dot{\omega }\times v-\omega \times \dot{v}\right)-2\left(\omega \cdot \dot{\omega }\right)\cdot \left(\omega \times v\right)\right]}{{∥\omega ∥}^{3}\cdot {∥\omega \times \dot{\omega }∥}^{2}}$$

The SAID is based on six scalar functions of time and it is aimed to provide a coordinate-free description of the relative motion between the two rigid bodies. The first three invariant parameters are angular velocities (*ω_1_−ω_3_*) representing the angular motion of the body around the ISA and of the ISA itself. The last three invariants are linear velocities (*v_1_−v_3_*) representing the translational motion of the body along the ISA and of the ISA itself [19]. Such a representation is worthy to be investigated, as it can be useful to model motor tasks and motion primitives, and can provide information independent of the CS chosen [8]. In addition to the SAID, the functional hinge angle was obtained by integrating the first invariant; i.e., the angular velocity around the ISA.

The estimation of the ISA is known to be unreliable for low angular velocities; and small rotations or angular velocity lead to larger errors in the estimation of the ISA [1,13,15,19]. In the limit case of the angular velocity being equal to zero, the ISA is not defined. As a consequence, the computation of the SAID is unreliable when the angular velocity is close to zero, which represents a singularity for the invariant features (Equations (7)−(11)). As suggested by previous studies, we assumed an angular velocity of 0.3 rad/s as the threshold value [15]. When the measured angular velocity was below the threshold, the parameters were not computed, and for visualization purposes, the samples of the SAID were replaced with zeroes.

### 2.4. Parameters

The parameters, detailed in the following, were computed twice: (i) based on the OS data and (ii) based on the inertial data. In the case of OS data, the angular velocity **ω** of the rigid body was obtained by differentiating the orientation matrix of the CS. The procedure consists of transforming the rotation matrix to its quaternion representation and then computing the rotational velocity using the derivative of the quaternion as in Equation (12), where the *q*_n_ values are the quaternion coefficients [28].

(12) $$\omega =2\left(\begin{array}{cccc} -q_{1} & q_{0} & q_{3} & -q_{2}\\ -q_{2} & -q_{3} & q_{0} & q_{1}\\ -q_{3} & q_{2} & -q_{1} & q_{0} \end{array}\right)\;\left(\begin{array}{c} {\dot{q}}_{0}\\ {\dot{q}}_{1}\\ {\dot{q}}_{2}\\ {\dot{q}}_{3} \end{array}\right).$$

In the case of MIMU sensors, the angular velocity was obtained as a direct measurement from the gyroscopes. The velocity $\dot{p}$ was computed as the derivative of the position vector of the CS. All the derivatives were computed using finite-differences. The data were smoothed using a recursive moving average filter [26] with a window of 10 samples, applied before and after computing the derivative.

**Figure 3:**
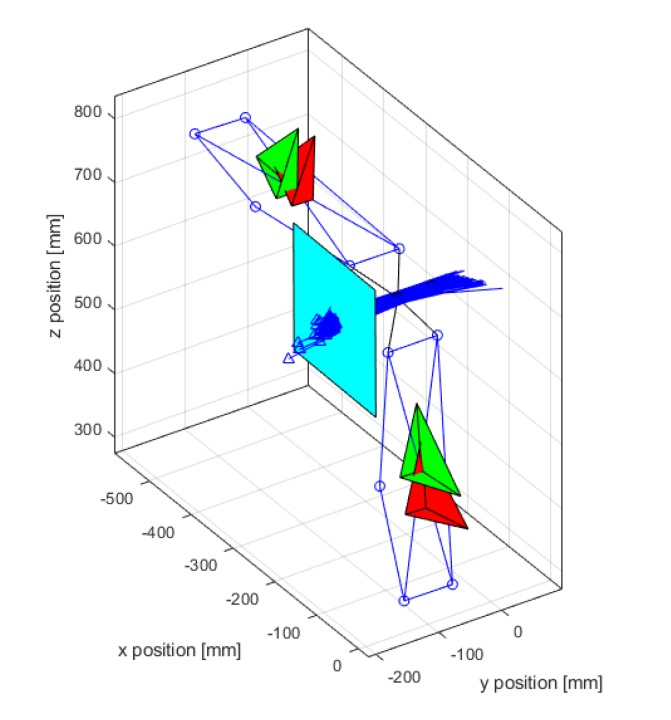
Reference plane for testing the variability in functional ISA axis estimation. The blue arrows intersecting the plane represent the instantaneous screw axes (ISA) computed for every sample of the trial.

In order to compare the results across the two systems, the following quantities were computed:

- Deviation angle *θ*: the average angular error between the direction of the functional ISA axis, represented by its unit vector ***n_f_*,** and the geometric axis, ***n_g_***, defined by the two markers at the extremities of the hinge. The angle was computed as the angle between two unit vectors in space (Equation (13)). The standard deviation (SD) of the deviation angle within the trial was computed as well.
  (13) $$\theta =\mathrm{acos}\left(n_{f}\cdot n_{g}\right)*\frac{180}{\pi }.$$
- Origin distance: the average linear distance between the geometric origin (midpoint of the two markers at the sides of the hinge joint) and the closest point on the ISA, ***s***, as in Equation (5). The SD of this distance was also computed.
- RMS difference: The root mean square value of the difference between the Euler angle around the *z*-axis, *φ_e_*, and the functional angle, *φ_f_*, computed as the integral of the *ω_1_* invariant (Equation (14)). The initial offset angle of the hinge was added to the integral, in order to make the curve comparable to the geometrical Euler angle (Equation (15)).
  (14) $$RMS\_diff=\sqrt{\frac{\sum_{i=1}^{N}{\left(\varphi_{f,i}-\varphi_{e,i}\right)}^{2}}{N}}$$
  (15) $$\varphi_{f}=\int^{\;}\omega_{1}dt+\varphi_{e}\left(t=0\right).$$
- RoM: The angular range of motion around the three axes of the joint. The angles *φ_e_*, *ϑ_e_* and *ψ_e_* represent the angular displacement of CS {2} with respect to CS {1} according to the Euler sequence *z-y-x.*
  (16) $$RoM_{z}=\left|\max\left(\varphi_{e}\right)-\min\left(\varphi_{e}\right)\right|$$
  (17) $$RoM_{Y}=\left|\max\left(\vartheta_{e}\right)-\min\left(\vartheta_{e}\right)\right|$$
  (18) $$RoM_{X}=\left|\max\left(\psi_{e}\right)-\min\left(\psi_{e}\right)\right|.$$
- In addition to the previous quantities, a virtual plane was defined perpendicular to the geometric ISA and at a distance of 10 cm from the geometric centre [29] (Figure 3). This allowed us to assess variability in the direction of the functional ISA, which ideally should be coincident to the geometric axis. The intersection of the functional ISA with this plane was analysed, while the rotation centre of the functional ISA was forced to be at the geometric centre of the hinge [29]. The variability of the ISA was then quantified by means of the confidence ellipse containing 95% of the points representing the intersection of the ISA with this plane.

To quantify the repeatability of the measurements across the different trials, the coefficient of variation (CV) was computed as the percentage ratio between standard deviation (SD) and the mean value of each quantity. In addition, the repeatability coefficient (CR) was computed by multiplying the SD by 2.77 [30]. The CR quantifies the absolute reliability of measurements; i.e., the value below which the absolute difference between two measurements would lie with 0.95 probability [30]. Furthermore, all the quantities were tested for significant differences between the OS and MIMU systems. Data groups were preliminary tested for normality by means of the Shapiro–Wilk test. When the data were found to be normally distributed, the paired t-test was applied. When the data did not follow a normal distribution, the non-parametric Wilcoxon’s signed rank test was applied.

All data processing was done in MATLAB™ (Mathworks, Natick, MA, USA). 

## 3. Results

**Figure 4:**
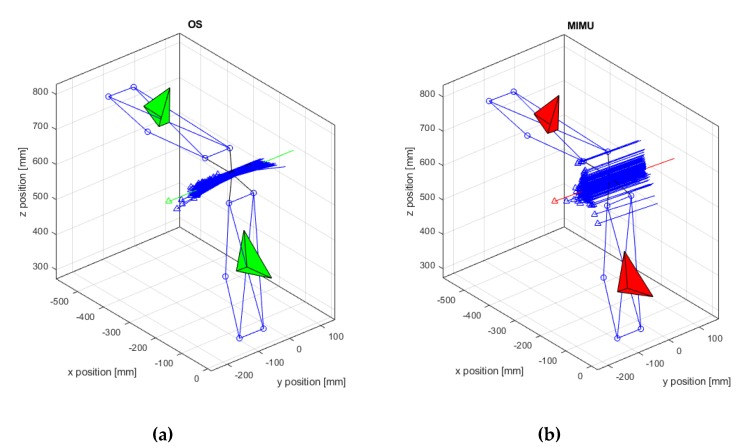
Estimation of the functional axis (**a**) OS data—green CS, and (**b**) MIMU data—red CS. The green and red arrows represent the mean ISA.

**Figure 5:**
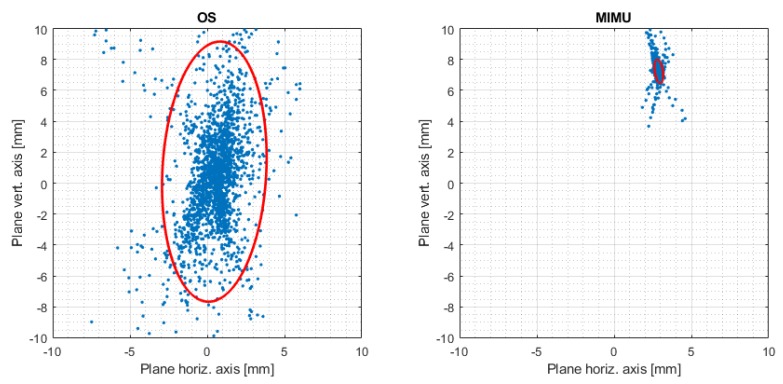
Confidence ellipse for 95% of points of intersection with the reference plane for one trial. Comparison of OS and MIMU. The centres of the graphs represent the geometrical axes.

**Figure 6:**
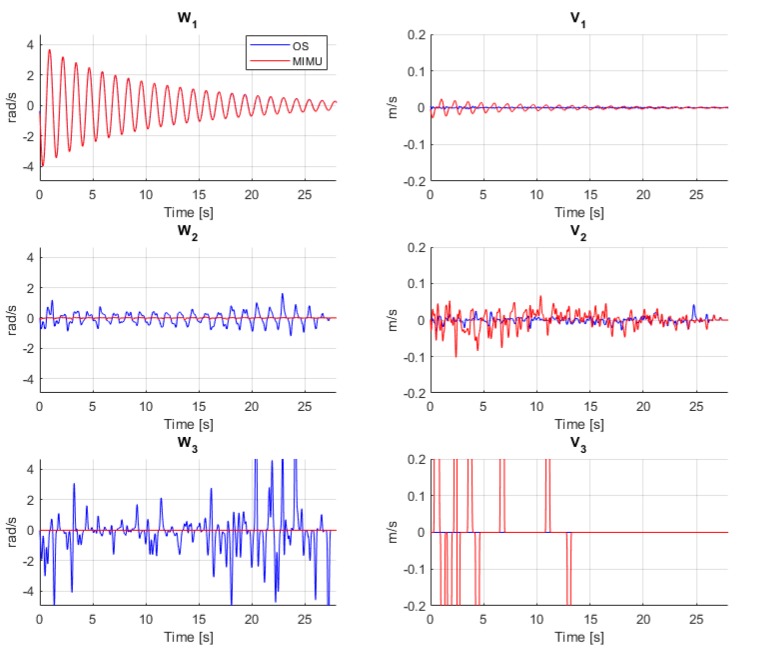
Screw axis invariant descriptor for one trial of the free swing motion of the hinge as computed by the OS (blue) and MIMU (red).

The results are presented in Table 1 and the following Figure 4, Figure 5 and Figure 6. The estimation of the ISA axis and its parameters by means of the OS and the MIMU system were compared. All the parameters showed significant differences between the two systems, except for the angular range of motion around the main axis of rotation. 

The CR and CV suggested a good repeatability for the ISA direction measurements with a CV < 6% for the OS and CV < 2% for the MIMU. A lower repeatability was observed for the computation of the origin in the MIMU case (Table 1). 

The ISA estimated by the MIMU had a larger deviation angle and a larger distance from the geometrical reference (Table 1). However, the standard deviation of the angle measured by the MIMU was lower than the OS. This result was confirmed by the smaller confidence ellipse observed for the MIMU (Figure 5). Furthermore, a systematic deviation from the reference, i.e., the central point of the plane, was observed (Figure 5). The larger ellipse observed for the OS suggests a larger dispersion from the average value that was closer to the reference. The centre of the plane, corresponding to the coordinates [0,0], represented the position of the geometrical axis. The ellipse axis lengths (last two lines of Table 1) were also significantly higher for the OS system. These results showed that the OS could determine the ISA more accurately but with a lower precision, while the MIMU had a higher precision but lower accuracy. In both cases, the highest variability was observed along the vertical direction (Figure 5). 

The SAID of the hinge motion is shown in Figure 6. The graphs represented the time window when the angular velocity was above the threshold of 0.3 rad/s. Since the motion was close to a pure rotation, the first invariant, *ω_1_*, correctly corresponds to the rotation around the ISA (first graph of first column in Figure 6). The second invariant, *ω_2_*, detected some angular variations for the OS system, while it was close to zero for the MIMU. The third invariant, *ω_3_*, presented some very large variations for the OS, while it was close to zero for the MIMU. The fourth invariant, *v_1_*, was close to zero in the OS case, while some small periodic motion was detected for the MIMU. The fifth invariant *v_2_*, also suggested some ISA displacements for the MIMU case, although it was affected by noise. The last invariant *v_3_* showed high oscillations but could not be considered reliable due to the high noise on the previous invariants.

## 4. Discussion

This work presents an experimental setup designed to record and analyse the rotational motion around a hinge by two different measurement systems: the OS (Vicon) and the MIMU (Xsens). The ISA, its parameters and the invariant representation of the hinge motion were computed based on the data recorded by the two systems. The experimental setup with two segments and two sensors was designed in order to give a first-order approximation of a human hinge-like joint, such as the knee. Such a setup can facilitate future application to a more advanced human model or in-vivo testing. 

The results clearly demonstrated a difference in performance between the two systems. The estimation of the direction of the ISA was more precise and more repeatable when based on inertial data. The ISA direction was, instead, more accurate when based on the OS data but with a lower precision, as shown by the confidence ellipse in Figure 5. The estimation of the ISA origin was less accurate and less precise when based on inertial data. Furthermore, a lower repeatability for the ISA origin was observed for the MIMU case. The poor precision in the ISA origin, obtained from the MIMU data, could be explained by the fact that its computation was based on the variation in space of the direction of the axis [15]. Given the good repeatability and high precision of the measurement of the ISA direction (angle), the poor accuracy could be attributed to the non-perfect alignment of their CSs due to the experimental setup that introduced an error in the measurements. In fact, in order to compare the angular velocities and the relative parameters, the outputs of the MIMUs needed to be expressed in the ground reference system; i.e., CS {0}.

We computed the coefficient of repeatability (CR) in order to index the measurement error. It takes into account both random and systematic errors in measurements [30]. The CR scores were lower for the MIMU case for most of the parameters, indicating a good repeatability of the measurements. Poor repeatability was observed for the origin of the ISA when measured by the MIMU.

The confidence ellipse allowed us to describe the variation of the ISA with respect to a virtual sagittal plane. This plane was defined according to recommendations of [29], as it proved to be a good reference for describing the motion of the ISA axis. The range of motion on the main rotation axis was comparable across the two systems, while a larger lateral motion was observed in the case of the MIMU. This is coherent with the previously discussed results and was attributed to the experimental error of the measurement. The ellipse size (Figure 5) and its parameters, i.e., the length of the axes (Table 1), confirmed the lower dispersion and higher precision of ISA measurements when computed by inertial data. The distance between the ellipse centre and the origin of the plane, i.e., point [0,0], shows the systematic measurement error previously discussed. The angular range of motion in the lateral directions, i.e., *x* and *y* directions in Table 1, should ideally be zero, as no motion occurs in these directions. Higher RoMs in these directions were observed in the case of MIMU data. These angles were relatively small when compared to the displacement around the hinge axis, and were attributed mainly to the measurement noise, artefacts and systematic errors introduced in the processing procedure.

Assuming the hinge motion as a pure rotational motion around the ISA, that ideally should coincide with the geometrical axis of the hinge, the SAID should contain information only in the first graph (i.e., *ω_1_*), while the other graphs representing translational motion along the hinge (*v_1_*) and displacements of the ISA *(ω_2_, ω_3_, v_2_* and *v_3_*) should be close to zero. The first invariant, *ω_1_*, correctly described the angular velocity around the ISA for both systems (Figure 6). The second invariant, *ω_2_*, that represents the lateral oscillation of the ISA, was close to zero for the MIMU system, reflecting the higher precision in the estimation of the ISA direction and making it closer to the ideal case. Instead, *ω_2_* had some fluctuations when computed with the OS data due to the noise/artefacts of the measurements amplified by the derivatives [19]. The fourth invariant, *v_1_*, was close to zero in the OS case, indicating no motion along the axis, coherently with the experimental setup that did not allow motion along this axis. Instead, some motion was detected for the MIMU, coherently with the effect of experimental error previously described. The fifth invariant, *v_2_*, also suggested no lateral displacement of the ISA, although it was affected by noise. The third and sixth invariants, *ω_3_* and *v_3_* (last row of Figure 6), showed high oscillations but could not be considered reliable, due to the noise amplification effect of the finite-differentiation procedures that, for those quantities, were applied twice [19]. The spikes observed in such graphs were attributed to the effect of noise amplification in differential kinematics and to the effect of repeated zero crossing due to the noise that affects the invariant computation [19]. It is worth remarking that all the data points corresponding to an angular velocity < 0.3 rad/s were removed from the analysis as it is known that they may produce large artefacts [15]. Such points were replaced with zeroes in the SAID.

The different performances of the two systems were attributed mainly to the fact that the angular velocity was obtained as a direct measurement in the case of MIMU sensors and as an indirect measurement in the case of the OS. It is in fact well known that the numerical differentiation procedure amplifies the noise [10,13,27] and may lead to artefacts or unreliable results in the computation of the ISA and invariants, especially at low angular velocities [15,19]. Based on the results of this experiment, it is clear that the direct measurement of angular velocity by means of gyroscopes improves the precision of the ISA direction estimation. However, it is known that inertial sensors lack a consistent ground reference system, and the measurement orientation can be affected by magnetic disturbance [31]. To study the relative motion of the two rigid bodies, it was necessary to reference the measured orientation to the same global reference system. For this purpose, it was assumed that the global reference of each sensor did not change across the measurements. Small variations due to magnetic phenomena are possible and may lead to inaccuracies [22]. According to the formulation of the invariant features, such inaccuracies affect mainly the invariants based on translational velocity.

In this study we investigated a simple case of relative hinge-like motion between two rigid bodies. Studying the screw axis invariant descriptor can simplify the biomechanical analysis of joints and the interpretation of results, especially regarding the relative motion of body segments; e.g., the shank and the thigh that are of great interest for gait analysis [32]. Since the SAID is not affected by the external referencing of data, it can be assumed to be a complete and coordinate-independent description of the motion. 

Previous studies already attempted to compute the ISA of the human knee facing issues, mainly related to soft tissue artefacts and measurement noise. E.g., an ex-vivo experiment had some OS markers rigidly attached to the bones, allowing an increase in accuracy of the ISA computation and the analysis of its typical variational pattern across a gait cycle [29]. A similar study was done in-vivo with OS markers rigidly fixed to the bone by means of intracortical pins [18]. Although such an approach can increase measurement accuracy, it cannot be adopted in the daily clinical practice, being highly invasive.

Another previous study comparatively examined the knee rotation axes computed in a functional way and a geometrical way [33]. The geometrical axis was based on magnetic resonance imaging, while the functional one was based on motion capture data collected during an isokinetic dynamometry measurement. The study determined that, for that specific task, the functional approach can describe the motion better than the geometric approach. The maximum angular difference was 10.6° and the maximum origin distance was 20.8 mm [33]. 

Our results (Table 1) can be compared to the study of Schwartz et al. [16], where: (i) the average distance between the measured functional centre and the geometric centre was 3.8 mm, (ii) the angular deviation of the functional axis from the geometric one was 2.0° and (iii) in general, it was observed that the approximation of the knee rotation centre was the least reliable parameter to be estimated by the functional approach.

Based on the results of the present study, it is recommended to compute the ISA direction and origin based on marker data. The estimation of ISA direction can be significantly improved by inertial measurements; however, further studies are required to identify and remove the errors observed. The invariants based on angular velocity were a good representation of the real-world scenario when based on inertial data. Instead, the linear velocity invariants were closer to the physical motion when based on OS data. Some solutions involving OS and MIMU data fusion should be studied in future works in order to improve both accuracy and precision of the measurements.

The findings discussed in the present paper were limited to our experimental setup that allowed the analysis of a pure-rotational motion on a well-controlled environment. While this remains a useful preliminary analysis about the computation of the ISA and the SAID, it may not accurately represent the complex motion of human body joints. For this reason, further studies are recommended to investigate more advanced cases, such as three-dimensional motion, human motor tasks or the perturbed motion of a mechanical system.

## 5. Conclusions

This study comparatively examined two measurement systems, the OS and the MIMU, for the calculations of the ISA and the SAID of a simple motion task based on a mechanical hinge setup.

The SAID provided useful information about the motion with respect to its ISA in terms of angular velocity around the ISA, translational velocity along the ISA and displacement of the ISA itself. Results from this study showed that the MIMUs were more precise and more repeatable but less accurate in estimating the ISA direction. They also had a lower accuracy and precision in estimating the ISA origin. Instead, the OS was more accurate for the direction and origin, but less precise.

The invariants based on angular velocity were better estimated by the MIMU, while the translational velocity invariants were better estimated by the OS. It is, therefore, recommended to use a combination of inertial and optoelectronic sensors to accurately compute the ISA and the full SAID of motion. As the computation of the ISA and invariants is affected by noise, it can take advantage of the direct measurement of the angular velocity by means of gyroscopes; thus, further studies are recommended to investigate and reduce the sources of the error observed. Future studies should also investigate the ISAs and the SAIDs for the anatomical joints of the human body.

## Figures and Tables

**Table 1:** Results for the computed parameters and comparison between OS and MIMU. Mean value, repeatability coefficient (CR) and coefficient of variation (CV) across the five repetitions of the task; * significant differences with *p* < 0.05.

	OS			MIMU			t-Test or Wilcoxon’s
	Mean	CR	CV [%]	Mean	CR	CV [%]	(*p*-Value)
Deviation Angle [°] *	2.17	0.36	5.92	4.37	0.22	1.83	<<0.01
SD of Deviation Angle [°] *	2.26	0.37	5.87	0.20	0.07	13.24	<<0.01
Origin Distance [mm] *	12.28	0.98	2.87	39.22	29.24	26.91	<0.01
SD of Origin Distance [mm] *	8.69	1.33	5.54	31.33	37.20	42.86	<0.05
RMS difference [°] *	0.69	0.19	9.99	0.61	0.07	4.05	<0.05
RoM_Z_ [°]	90.00	13.15	5.28	89.91	12.89	5.18	0.25
RoM_Y_ [°] *	0.50	0.47	33.74	4.58	0.65	5.14	<<0.01
RoM_X_ [°] *	1.32	0.36	9.84	3.30	0.73	7.98	<<0.01
Ellipse Axis 1 [mm] *	9.54	2.06	7.80	0.79	0.31	14.29	<<0.01
Ellipse Axis 2 [mm] *	3.18	0.62	7.00	0.29	0.10	12.24	<<0.01
